# Measuring internalized health-related stigma across health conditions: development and validation of the I-HEARTS Scale

**DOI:** 10.1186/s12916-024-03661-z

**Published:** 2024-10-08

**Authors:** Rebecca L. Pearl, Yulin Li, Laurie C. Groshon, Marian Hernandez, Danielle Saunders, Miriam Sheynblyum, Kimberly A. Driscoll, Joel M. Gelfand, Preeti Manavalan, Marjorie Montanez-Wiscovich, Deidre B. Pereira, Rebecca M. Puhl, Thomas A. Wadden, Lori B. Waxenberg, Sarah C. Westen, Xiang-Yang Lou

**Affiliations:** 1 Department of Clinical and Health Psychology, College of Public Health and Health Professions, University of Florida, Gainesville, FL USA; 2 Department of Biostatistics, College of Medicine and College of Public Health and Health Professions, University of Florida, Gainesville, FL USA; 3 Department of Psychology, San Diego State University, San Diego, CA USA; 4 Department of Dermatology and Department of Biostatistics, Epidemiology and Informatics, Center for Clinical Sciences in Dermatology, University of Pennsylvania Perelman School of Medicine, Philadelphia, PA USA; 5 Department of Medicine, College of Medicine, University of Florida, Gainesville, FL USA; 6 Department of Dermatology, College of Medicine, University of Florida, Gainesville, FL USA; 7 Department of Human Development & Family Sciences, University of Connecticut, Storrs, CT USA; 8 Center for Weight and Eating Disorders, Department of Psychiatry, University of Pennsylvania Perelman School of Medicine, University of Pennsylvania, Philadelphia, PA USA

**Keywords:** Cancer, Chronic disease, Diabetes, HIV, Internalized stigma, Obesity, Pain, Psychometrics, Skin disease

## Abstract

**Background:**

Health-related stigma and its internalization among individuals with chronic health conditions contribute to impaired mental and physical health and quality of life. Research on health-related stigma has been siloed, with disease-specific measures that may not capture the experiences of individuals with multiple health conditions and that prevent comparisons across health conditions. The current study aimed to develop and test a transdiagnostic measure of internalized health-related stigma for use among adults with different physical health conditions.

**Methods:**

An existing measure of internalized mental health stigma was adapted to assess stigma due to chronic physical health conditions following COSMIN procedures, with input from advisory boards of community members living with a range of stigmatized health conditions (obesity, type 1 and type 2 diabetes, skin diseases, HIV, chronic pain, and cancers) and of health professionals who specialized in these conditions. The new Internalized Health-Related Stigma (I-HEARTS) Scale was tested in an online sample of 300 adults with these health conditions, recruited from ResearchMatch. Additional psychosocial measures of mental health and quality of life were administered, and participants provided information about their health conditions and demographic characteristics. Exploratory factor analysis and tests of reliability and validity were conducted to determine the psychometric properties of the I-HEARTS Scale, and k-means clustering and receiver of characteristic curve analysis were used to determine a clinically meaningful cutoff score indicating high levels of internalized stigma.

**Results:**

Factor analysis results yielded a 25-item scale with a 3-factor solution, with subscales of Perceived and Anticipated Stigma, Stereotype Application and Self-Devaluation, and Stigma Resistance. Psychometric properties for internal consistency, inter-item and item-total correlations, and test-retest reliability were strong. Certain demographics (e.g., younger age) and characteristics related to health conditions (e.g., greater symptom severity) were associated with higher levels of internalized stigma. I-HEARTS Scale scores correlated moderately to strongly with related but distinct psychosocial measures, and a cutoff score of 3.40 or higher on the 1–7 rating scale was determined to indicate clinically meaningful levels of internalized stigma.

**Conclusions:**

The I-HEARTS Scale is a reliable and valid measure for the assessment of internalized health-related stigma among adults with varied stigmatized chronic health conditions.

**Study pre-registration:**

https://osf.io/84c5d/?view_only=87238512f6d6475c87f8f64280a8a15f.

**Supplementary Information:**

The online version contains supplementary material available at 10.1186/s12916-024-03661-z.

## Background

Individuals with chronic health conditions commonly face negative judgment, social rejection, mistreatment, discrimination, and blame for their illness [1]. This stigmatization occurs across a range of health conditions such as obesity [2], type 1 and type 2 diabetes [3], skin diseases [4, 5], HIV [6], chronic pain [7], and cancers [8]. These and other health conditions (including mental health conditions) vary in key characteristics, such as whether or not they are considered to be preventable or controllable (and, thus, blamed on individuals), how visible versus concealable they are to others, and their perceived contagiousness. Despite these differences, the umbrella construct of *health-related stigma* has emerged in response to documented consistencies across health conditions in the manifestations and impacts of stigma [9–12]. For example, across a wide range of both mental and physical health conditions, stigma undermines management of health conditions, exacerbates symptoms, and adds further health risks through pathways that include: heightened stress; engagement in unhealthy coping behaviors; reduced engagement in disease management behaviors (e.g., taking medication); diminished access to resources (e.g., due to discrimination); avoidance of health care; poorer quality treatment received in health care; and impaired mental health [1, 13]. Pachankis et al. [14] conducted a cluster analysis of 93 stigmatized traits and found that many mental and physical health conditions clustered together on the basis of shared stigma dimensions (e.g., persistence, disruptiveness). Comorbidity among health conditions is also common (including co-occurrence of mental and physical health conditions [15–20]), further supporting the value of a holistic approach to the study of health-related stigma.

Due to pervasive negative societal attitudes and experiences of being stigmatized by others, many individuals with chronic health conditions internalize these negative attitudes and devalue themselves because of their health conditions (i.e., self-stigmatize) [21–26]. A prominent model of internalized stigma—developed for mental health stigma but also applied to and tested in studies of physical health stigma [27–31]—describes a stepwise process of (1) awareness of stereotypes and stigma based on one’s identity or traits; (2) agreement with negative stereotypes and stigmatizing attitudes; (3) self-application of stereotypes and stigma; and (4) diminished self-esteem [22]. This model also asserts that internalized stigma undermines self-efficacy, or confidence in one’s ability to pursue general life goals and goals related to self-management of health conditions [32, 33]. Additional factors included in the conceptualization of internalized stigma and its consequences are shame or embarrassment about one’s health condition(s) and social withdrawal or isolation to avoid judgment or stigmatizing interactions [29, 30, 34–41]. Across different health conditions, internalized stigma is linked to increased stress, depression, and anxiety, and reduced mental and physical health-related quality of life (HRQOL) [6, 23, 42, 43]. Growing recognition of the harms of internalized health-related stigma has led to increased calls for interventions to prevent and reduce it [1, 12].

Disease-specific self-report measures of internalized stigma have been developed and tested across an array of health conditions [12, 24, 25, 44, 45]. These are useful tools that capture the unique experiences and concerns of individuals with each type of health condition. However, disease-specific questionnaires are limited in their ability to capture stigma internalized by individuals with multiple chronic health conditions, which represents the majority of people with chronic illness [46]. This may be especially true if individuals cannot distinguish how each health condition affects their thoughts and feelings about themselves, as they may instead experience the effects of stigma more globally.

Furthermore, disease-specific measures cannot be used in studies that aim to identify similarities and differences in stigma across multiple health conditions, since direct comparisons would require use of a single measure. Such comparisons may be useful to enhance theoretical understanding of internalized stigma and to identify possible cross-cutting targets for intervention [12]. Future efforts to design and test generalizable (rather than disease-specific) interventions for internalized health-related stigma could potentially enhance dissemination and implementation [12]; for example, a “transdiagnostic” counseling or peer support intervention for internalized stigma could be accessed by patients with many types of health conditions within a health system, rather than requiring individual clinics or specialties to develop and deliver their own disease-specific interventions. Investigating generalizable interventions requires a measure of internalized stigma that can be administered to individuals with different kinds of health conditions. Prior research has identified common elements across disease-specific measures of internalized stigma [11, 12] but has not developed and tested a measure for the purpose of assessing internalized health-related stigma more broadly.

The current study aimed to develop and validate a transdiagnostic measure of internalized health-related stigma that can be administered to adults with a range of different chronic physical health conditions. Specific health conditions included in the current study were obesity (defined based on the current World Health Organization definition of a body mass index of 30 kg/m^2^ or higher); type 1 and type 2 diabetes; skin diseases (e.g., psoriasis, eczema, vitiligo); HIV; chronic pain; and cancers. These conditions were chosen because they represent a range of visible and invisible conditions, conditions that are and are not generally viewed as “preventable,” and conditions that vary in their perceived contagiousness. Thus, these conditions, while all stigmatized, cut across different dimensions of stigma such as blame, concealability, and contagion. The focus of this work was primarily on physical health stigma, given prior extensive research on the assessment of mental health stigma across different mental disorders [47]. Recent theoretical models of health-related stigma highlight commonalities across different forms of stigma and emphasize the need for overarching solutions [1, 12]. The Internalized Health-Related Stigma (I-HEARTS) Scale was developed to advance research that can leverage knowledge across health fields and promote collaboration and cross-disciplinary research to understand and reduce health-related stigma.

## Methods

### Scale development

The scale development process followed the COSMIN methodology for assessing content validity of patient-reported outcome measures [48], with some modifications. Initial items were adapted from the Internalized Stigma of Mental Illness (ISMI) Scale [47, 49], which is widely used and validated in 50 countries. The ISMI Scale includes five subscales that capture specific domains of internalized stigma: Alienation; Stereotype Endorsement; Discrimination Experience; Social Withdrawal; and Stigma Resistance (with reverse-coded items). Although predominantly used to assess internalized stigma due to mental illness, prior work has adapted the ISMI Scale to assess stigma due to physical health conditions as well. The 28-item version of the ISMI Scale that has been validated and tested in samples of adults with physical health conditions (e.g., leprosy) [47, 50–52] was used as a starting point for scale development in this study.

Two advisory boards were involved in the I-HEARTS Scale development. The first board (Health Professional Advisory Board) was comprised of 11 researchers and clinicians with expertise on obesity, type 1 or type 2 diabetes, skin diseases, HIV, chronic pain, or cancers. The second board (Community Advisory Board) was comprised of 12 adults with lived experience with these health conditions. Board members were recruited nationally through professional networks and advocacy and community organizations. Efforts were made to recruit diverse board members of varying ages, genders, races, and ethnicities (see Supplementary Table S1 for Advisory Board member demographic characteristics).

An initial draft of the I-HEARTS Scale, with items from the 28-item ISMI Scale (including all subscales) [47], was circulated to Board members. Small virtual group meetings (separated into Health Professional and Community Advisory Board meetings, respectively) were held to obtain feedback on the items, instructions, and rating scale. Changes were made based on this feedback, including editing the wording of items, adding new items, and removing items that were not considered relevant.

After initial feedback was incorporated, an updated version of the scale was circulated with a formal online Qualtrics survey for Board members to complete. The survey questions followed COSMIN guidelines [48] by asking Board members to rate the clarity and relevance to their health condition(s) of expertise of each item on a scale from 0 to 2 (0 = not at all, 1 = somewhat, 2 = very). They were also asked to recommend whether each item should be rejected, accepted with modification (with accompanying suggestions), or accepted. Board members were asked about the comprehensiveness of the measure, relevance of all domains captured by the measure’s subscales, and the order in which items were presented. Formal feedback on the scale instructions and rating system was also obtained.

Feedback from this survey (see Supplemental Table S2) informed final modifications to produce a 30-item scale with response ratings from 1 (strongly disagree) to 7 (strongly agree), which was then tested in a validation study; after testing, results were circulated to Board members for final comments and approval of the scale. Thus, Board members were involved in every step of the scale development process to enhance construct validity (i.e., to ensure that the measure was capturing internalized stigma as experienced by adults with different types of chronic health conditions), and to ensure that stigma was not inadvertently perpetuated in scale items.

### Scale validation

#### Participants

An online survey of 300 adults with chronic physical health conditions was conducted to test the I-HEARTS Scale’s psychometric properties, to select the final set of scale items, and to test for validity and reliability. Survey participants were recruited from ResearchMatch, which is a national health volunteer registry created by several academic institutions and supported by the US National Institutes of Health as part of the Clinical Translational Science Award program. ResearchMatch has a large population of volunteers who have consented to be contacted by researchers about health studies for which they may be eligible. ResearchMatch participants provide information about their demographic characteristics and health conditions, and researchers can use a random selection tool to invite participants with specific characteristics to receive more information about their study. ResearchMatch participants can agree or decline to receive this information.

#### Procedures and measures

ResearchMatch participants with obesity, type 1 or type 2 diabetes, skin diseases, HIV, chronic pain, or cancers were randomly selected to receive study invitations between May and September 2023. The study was advertised as being about “health-related thoughts and social experiences” (the term “stigma” was not used). Prespecified benchmarks were set to recruit at least 50 participants per category of health condition, and efforts were made to oversample participants from minority racial and ethnic backgrounds. Those who agreed to receive more information about the study were directed to the online survey, hosted in REDCap, of which the first page was the study’s informed consent form. Participants were required to select a box indicating that they agreed to participate in order to proceed with the survey.

After consenting, participants were directed to a screening questionnaire to determine their eligibility. To be eligible for the study, participants had to be 18 years or older (indicated by self-reported age and computed from month and year of birth). Participants were also required to indicate that they had a current health condition that fell into at least one of the six categories described above (additional physical or mental health conditions may have been present as well). For participants who indicated that they had “obesity or high body weight,” follow-up questions asked their current height and weight to compute body mass index. Participants who endorsed skin disease, chronic pain, or cancer were asked to specify their diagnosis (or the location of their pain). Participants with cancer were eligible regardless of whether their cancer was active or in remission. Participants were also asked additional questions about their health conditions, such as their duration and symptom severity. Those who were ineligible based on their responses were automatically directed to an ineligibility message. Eligible participants were then asked to rate their overall symptom severity across all health conditions; whether any of their health conditions were visible to others (with response options of “no,” “yes, sometimes,” and “yes, all of the time”); whether they had ever been “viewed differently, judged, blamed, or treated poorly by others” (i.e., stigmatized) because of their health conditions; and to rate how much their health conditions affected how they thought and felt about themselves on a scale from 1 (not at all) to 5 (extremely).

Participants then completed the I-HEARTS Scale, followed by several relevant measures of mental health and quality of life to determine convergent and discriminant validity. These measures included the 30-item Internalized Shame Scale (with subscales for Shame and Self-Esteem) [52–55] and the 20-item UCLA Loneliness Scale (version 3; which also captures social isolation) [56]. Both scales are widely used with strong psychometrics and capture constructs highly relevant to and correlated with internalized stigma [29, 30, 34–41]. Participants also completed the 6-item Emotional Representations subscale of the Revised Illness Perceptions Questionnaire, which assesses the emotional impact of a person’s health condition [57]. Given prior evidence of the relationship of internalized stigma with self-efficacy and stress [11, 32, 33], participants completed the 10-item General Self-Efficacy Scale [58] and the 4-item Perceived Stress Scale [59, 60] To establish the clinical significance of scale ratings, participants completed the Patient Health Questionnaire-9 (PHQ-9; to assess depression) [61]; the Generalized Anxiety Disorder-7 (GAD-7; to assess generalized anxiety) [62]; the 10-item Severity Measure for Social Anxiety Disorder [63]; and two measures of mental and physical HRQOL (four items from the Centers for Disease Control [CDC] Healthy Days Core Measure [64] and the 12-item World Health Organization Disability Assessment Schedule [WHODAS] 2.0 [65]). The CDC Healthy Days Core Measure assesses overall self-rated health and the number of days in the past month of poor mental and physical health (i.e., unhealthy days), and the WHODAS 2.0 assesses impaired functioning due to health conditions. Participants also were asked to report demographic characteristics such as age, sex, gender, race, ethnicity, sexual orientation, education, employment, income, and marital status.

A small subset of participants was invited to complete the I-HEARTS Scale again 2 weeks later to determine test-retest reliability, with a goal of obtaining 50–60 responses [66, 67] and at least 10 responses from participants with each of the six types of health conditions. Pre-determined benchmarks were also set for gender, race, and ethnicity, and efforts were made to have adequate representation across participant age and overall symptom severity. Participants were invited consecutively to the follow-up survey until benchmarks were met. Participants were debriefed after their participation was complete (those who were selected for the 2-week follow-up survey were debriefed after completing the second survey). Debriefing included a list of resources for participants to learn more information about stigma related to different health conditions. Participants were compensated with an electronic gift card of their choosing and received $15 for completing the main survey, and an additional $10 if they completed the 2-week follow-up survey. The institutional review board approved all study procedures. Study procedures were preregistered in Open Science Framework: https://osf.io/84c5d/?view_only=87238512f6d6475c87f8f64280a8a15f.

#### Data quality and inspection

Participants were given 1 week to complete the main survey and the 2-week follow-up survey. Those with missing items were contacted to provide responses. Participants were excluded if they did not complete at least two-thirds of the main survey questionnaires, or if they had any items missing on the I-HEARTS Scale.

Initial data quality checks included checking for implausible values of height, weight, and body mass index (for participants who provided this information); discrepancies in age, based on comparing self-reported age with calculated age using reported birth month and year; suspicious patterns of responses, such as selecting the same response rating for every item on one or more scales (including for reverse-scored items) or zigzag patterns of item responses; and completing the survey in less than 10 min.

After initial data collection and quality inspection, additional types of responses of questionable validity were observed (e.g., in optional free response items). Upon further inspection, discrepancies were found between demographic characteristics and health conditions reported in the survey versus those that participants had provided in ResearchMatch. In response to this discovery, procedures were changed such that key demographic characteristics were added to the screening questionnaire, and all screening responses were manually checked in real time against information in ResearchMatch (rather than allowing participant to automatically continue to the survey). Participants with discrepant information or other suspicious responses (e.g., long lists of health conditions in ResearchMatch that would not be plausible for one person to have) were not eligible to continue with the survey. Those with consistent information were sent a separate link to continue to the rest of the survey. Additional data quality checks were included, such as: a Captcha at the start of the survey; a required free response item; an attention check item; and duplicate items for month and year of birth (in the screening survey and again in the main survey). Participants were excluded if they failed any of these data quality checks.

#### Data analyses

After participant exclusions were made, data quality and integrity were further checked by assessing the data for missing and out-of-range values with basic statistical procedures, including univariate statistics and visual graphical displays (e.g., scatter plots). Minimal missing data was observed. Descriptive statistics were computed for participant characteristics and for the I-HEARTS Scale, including item-level and total mean, median, standard deviation, and range. Scale psychometrics were computed, including: exploratory factor analysis to determine factor loadings of each item and separation into subscales; Cronbach’s alpha (for internal consistency); inter-item and item-total correlations; and test-retest reliability for the subset of participants who completed the scale again 2 weeks later. Post hoc confirmatory factor analyses were also conducted to assess measurement invariance across health condition categories and across participants with one or multiple health conditions.

Correlations, analyses of variance (ANOVAs), and linear regression models were used to determine if differences in I-HEARTS Scale scores emerged in relation to participant characteristics, including categories of health conditions. Correlations were used to test for convergent and discriminant validity of the I-HEARTS Scale with the other included measures. Residual analyses were conducted to check for outliers and influential points and violations in the normality assumption (using Shapiro-Wilk test). If violations were detected, variance-stabilizing transformations (Box-Cox, log, square root, and inverse transformations) or non-parametric tests (e.g., Spearman’s rank correlation and Kruskal-Wallis test) were used.

In addition, a cutoff score on the I-HEARTS Scale was determined with the k-means clustering technique (one of unsupervised learning methods) without reference to an external measure, and with the receiver of characteristic (ROC) curve analysis (e.g., that with highest Youden index) with reference to validated cutoff scores on the PHQ-9 and GAD-7 (a cutoff score of 10 or above was used to indicate moderate or greater severity of depression and anxiety, respectively [61, 62]). The PHQ-9 and GAD-7, which are widely used and recommended to assess depression and anxiety in medical populations [68–70], were chosen as external reference measures in order to establish a clinically meaningful cutoff score. After a cutoff score was selected, its clinical utility was tested by determining whether the cutoff score differentiated participants with high versus low depression and anxiety (using ANOVA for continuous PHQ-9 and GAD-7 scores and logistic regression for dichotomized variables using PHQ-9 and GAD-7 cutoff scores of 10 or above). Statistical inferences were conducted at the two-tailed significance level of 0.05, and analyses were implemented with SAS (version 9.4, Cary, NC, USA) and R software (version 4.3, R Development Core Team, Vienna, Austria).

## Results

Invitations were sent to 5544 ResearchMatch participants, of which 808 agreed to be contacted about the study, 733 consented and completed the screening questionnaire, and 506 completed the main survey. Overall, 389 individuals were excluded based on screening criteria and data quality checks; 42 participants did not start or discontinued the main survey; one participant had a missing item on the I-HEARTS Scale; and one participant withdrew from the study. Compared to included participants, those who were excluded due to poor data quality were younger and more likely to report their sex and gender as male and their race as Black or African American; however, many exclusions were based on discrepancies in demographic characteristics reported by respondents in the survey versus in their ResearchMatch profiles (e.g., identifying as Black in the survey but white in ResearchMatch), so true demographic differences between included and excluded participants cannot be determined.

After exclusions, a total of 300 participants were included in the study. Of the eligible participants, 64 were invited to complete the 2-week follow-up survey, and 55 completed it. Participant characteristics are displayed in Table 1, with additional details of specific health conditions summarized in Supplemental Table S3. Participants were predominantly non-Hispanic white, women, of middle age, and reported multiple chronic health conditions, with mild to moderate severity of symptoms.

**Table 1:** Participant characteristics

Variable	*N* (%) or mean ± standard deviation
Health condition	
Obesity	121 (40.3%)
Diabetes	95 (31.7%)
Type 1	35 (11.7%)
Type 2	60 (20.0%)
Skin disease^a^	90 (30.0%)
HIV	49 (16.3%)
Chronic pain^a^	113 (37.7%)
Cancer^a^	74 (24.7%)
One of the above types of health conditions	128 (42.7%)
Two types of health conditions	117 (39.0%)
Three or more types of health conditions	55 (18.3%)
Overall symptom severity	
No symptoms or health consequences	15 (5.0%)
Minimal	56 (18.7%)
Mild	80 (26.7%)
Moderate	119 (39.7%)
Severe	30 (10.0%)
Age	52.2 ± 16.7
Younger than 40 years	75 (25.0%)
40–60 years	117 (39.0%)
Older than 60 years	108 (36.0%)
Sex	
Female	198 (66.0%)
Male	100 (33.3%)
Not reported or missing	2 (0.7%)
Gender	
Female	191 (63.7%)
Male	93 (31.0%)
Nonbinary or fluid	9 (3.0%)
Transgender	2 (0.7%)
Genderqueer	3 (1.0%)
Prefer not to answer	2 (0.7%)
Race	
American Indian or Alaska Native	2 (0.7%)
Asian	14 (4.7%)
Black or African American	42 (14.0%)
White	214 (71.3%)
More than one race	18 (6.0%)
Other	6 (2.0%)
Prefer not to answer or do not know	4 (1.3%)
Ethnicity	
Hispanic or Latino/a	41 (13.7%)
Not Hispanic or Latino/a	255 (85.0%)
Prefer not to answer or missing	4 (1.3%)
Sexual orientation	
Heterosexual or straight	196 (65.3%)
Gay	42 (14.0%)
Lesbian	8 (2.7%)
Bisexual	25 (8.3%)
Pansexual	10 (3.3%)
Asexual	9 (3.0%)
Questioning or other	7 (2.3%)
Prefer not to answer or missing	3 (1.0%)
Years of education	16.2 ± 2.8
Employment status	
Employed full-time	106 (35.3%)
Employed part-time	32 (10.7%)
Unemployed	26 (8.7%)
Retired	74 (24.7%)
Not working due to disability	37 (12.3%)
Student	12 (4.0%)
Homemaker	9 (3.0%)
Prefer not to answer or missing	4 (1.3%)
Household income	
Less than $10,000	23 (7.7%)
$10,000–$49,999	94 (31.3%)
$50,000–$99,999	78 (26.0%)
$100,000–$149,999	38 (12.7%)
$150,000 or more	43 (14.3%)
Prefer not to answer or missing	24 (8.0%)
Marital status	
Married	121 (40.3%)
Live with partner	30 (10.0%)
Single (never married)	84 (28.0%)
Divorced or separated	43 (14.3%)
Widowed	14 (4.7%)
Other, prefer not to answer, or missing	8 (2.7%)

^a^Additional details related to health conditions in the categories of skin diseases, chronic pain, and cancers are presented in Supplemental Table S1

### Factor analysis

Item-level descriptive statistics of the I-HEARTS Scale are presented in Table 2. Parallel analysis with all 30 items using polychoric correlations indicated a three-factor solution that accounted for 61.2% of the variance. Table 3 displays results of the factor analysis using pattern coefficients of promax rotation with a three-factor solution. Factor analysis with the 30-item scale (Supplemental Table S4) resulted in items 7, 9, 11, and 26 being removed due to strong cross-factor loadings (difference ≤ 0.15 between factor loadings) and/or factor loadings <0.50 [71–73]. This procedure was repeated a second time with a 26-item scale, resulting in the removal of item 10. Parallel analysis and factor analysis were repeated for the 25-item scale and confirmed a 3-factor solution accounting for 64.0% of the variance, with all items retained (Table 3). Post hoc confirmatory factor analyses found strict measurement invariance across health condition categories and across individuals with one or multiple health conditions, further supporting the scale’s factor structure (see Supplemental Table S5).

**Table 2:** I-HEARTS Scale item-level descriptive statistics

Item	Mean ± SD	Median (Range)
1. **I feel out of place in most situations because of my health condition(s)**	3.48 ± 2.01	3 (1–7)
2. **Having this health condition(s) has ruined my life**	2.98 ± 1.96	2 (1–7)
3. **People without my health condition(s) could not possibly understand me**	4.08 ± 1.87	4 (1–7)
4. **I am embarrassed or ashamed that I have this health condition(s)**	3.58 ± 2.16	4 (1–7)
5. **I am disappointed in myself for having this health condition(s)**	3.24 ± 2.21	3 (1–7)
6. **I blame myself for my health condition(s)**	3.26 ± 2.13	3 (1–7)
7. I feel that I am a lesser person compared to others who don’t have my health condition(s)	3.13 ± 2.02	3 (1–7)
8. **I think that negative stereotypes (or assumptions) about people with my health condition(s) are true of me**	2.60 ± 1.77	2 (1–7)
9. I don’t think that people with my health condition(s) can live a good, rewarding life	2.31 ± 1.59	2 (1–7)
10. I don’t think I can contribute as much to society as other people can because of my health condition(s)	2.75 ± 1.97	2 (1–7)
11. I don’t think I’m appealing as a romantic partner because of my health condition(s)	4.17 ± 2.10	4 (1–7)
12. **People treat me unfairly because I have this health condition(s)**	2.99 ± 1.80	3 (1–7)
13. **Other people think that I can’t achieve much in life because of my health condition(s)**	2.83 ± 1.77	2 (1–7)
14. **People ignore me because I have this health condition(s)**	2.62 ± 1.80	2 (1–7)
15. **People take me less seriously because I have this health condition(s)**	2.80 ± 1.92	2 (1–7)
16. **Other people don’t believe that my health condition(s) affects me as much as I say**	4.05 ± 2.05	4 (1–7)
17. **Most people would not be interested in getting close to me because I have this health condition(s)**	2.95 ± 1.88	2 (1–7)
18. **If employers knew I had this health condition(s), they would be less likely to hire me**	3.76 ± 2.15	4 (1–7)
19. **I don’t share much about myself with others because I don’t want to burden them with my health condition(s)**	3.93 ± 2.03	4 (1–7)
20. **I don’t spend much time with other people because I worry what they will think of me due to my health condition(s)**	2.75 ± 1.95	2 (1–7)
21. **Negative assumptions about people with my health condition(s) keep me isolated from the world around me**	2.93 ± 2.03	2 (1–7)
22. **I stay away from social situations in order to protect my family or friends from feeling embarrassed by my health condition(s)**	2.52 ± 1.88	2 (1–7)
23. **Being around people who don’t have my health condition(s) makes me feel like I don’t belong**	2.92 ± 1.98	2 (1–7)
24. **I try not to get close to people who don’t have my health condition(s) to avoid rejection**	2.48 ± 1.89	2 (1–7)
25. **I don’t tell other people that I have this health condition(s) because I’m afraid they will judge me**	3.40 ± 2.13	3 (1–7)
26. ^a^I feel comfortable being seen in public with a person who has the same health condition(s) as me	2.83 ± 1.87	2 (1–7)
27. ^a^**In general, I am able to live life the way I want to even with my health condition(s)**	3.58 ± 1.84	3 (1–7)
28. ^a^**I can have a good fulfilling life, despite my health condition(s)**	3.06 ± 1.70	3 (1–7)
29. ^a^**People with my health condition(s) make important contributions to society**	2.22 ± 1.44	2 (1–7)
30. ^a^**Living with my health condition(s) has made me a strong person**	3.10 ± 1.72	3 (1–7)

Participants were instructed to “think about their health condition(s)” and rate their agreement with the statements based on their “thoughts and feelings in the past month.” *SD*, standard deviation. Items retained in the final scale are in bold

^a^Items 26–30 were reverse-scored

**Table 3:** Factor analysis of 25-item I-HEARTS Scale

Item	Factor 1	Factor 2	Factor 3	Communality
1	**0.66**	0.18	0.10	0.69
2	**0.65**	0.07	0.20	0.65
3	**0.85**	−0.21	−0.01	0.56
4	0.27	**0.68**	0.05	0.78
5	0.06	**0.89**	0.00	0.86
6	−0.16	**0.99**	−0.02	0.82
8	0.25	**0.56**	0.05	0.57
12	**0.86**	0.06	−0.08	0.73
13	**0.78**	−0.01	0.06	0.64
14	**0.73**	0.18	−0.02	0.71
15	**0.91**	−0.01	−0.05	0.78
16	**0.87**	−0.15	−0.08	0.59
17	**0.76**	0.20	0.00	0.78
18	**0.78**	−0.09	0.07	0.57
19	**0.61**	0.23	−0.11	0.52
20	**0.74**	0.18	0.10	0.82
21	**0.72**	0.24	0.04	0.82
22	**0.60**	0.38	0.02	0.78
23	**0.76**	0.15	0.03	0.76
24	**0.57**	0.40	−0.00	0.75
25	**0.54**	0.27	−0.04	0.51
27	0.25	−0.12	**0.81**	0.80
28	0.23	−0.06	**0.84**	0.86
29	0.06	−0.04	**0.80**	0.65
30	−0.45	0.24	**0.82**	0.67

Table displays pattern coefficients using polychoric correlations from factor analysis with promax rotation. Coefficients for items that were included in each factor are in bold. Items 27–30 were reverse-scored. Factor 1 sum of squares (SS) loadings: 13.06, proportion of the variance explained: 0.52; factor 2 SS loadings: 8.70, variance: 0.35, factor 3 SS loadings: 5.80, variance: 0.23

Table 4 displays total scale and subscale Cronbach’s alpha, average inter-item correlations, and average item-total correlations (Supplemental Table S6 displays these correlations for each item). Two-week test-retest reliability for the full scale using Lin’s concordance correlation coefficient was 0.80. Subscale test-retest reliability scores were as follows: factor 1 = 0.78; factor 2 = 0.69; factor 3 = 0.65.

**Table 4:** Total scale and subscale psychometrics for internal consistency

	Cronbach’s alpha	Average inter-item correlation	Average item-total correlation
Total scale	0.96	0.52	0.70
Subscales			
Factor 1: Perceived and Anticipated Stigma	0.96	0.63	0.78
Factor 2: Stereotype Application and Self-Devaluation	0.87	0.69	0.75
Factor 3: Stigma Resistance	0.82	0.59	0.68

Polychoric correlations were used for inter-item and item-total correlations. Factor 3 items were reverse-scored

Overall, the 25-item scale and its three subscales had acceptable to strong psychometrics and were used in subsequent analyses. The three subscales were labeled as: Perceived and Anticipated Stigma (factor 1); Stereotype Application and Self-Devaluation (factor 2); and Stigma Resistance (factor 3, consisting of reverse-scored items). Correlations between subscale scores and the total score were moderate to strong (Spearman’s *rho* for factor 1 = 0.97, factor 2 = 0.79, and factor 3 = 0.62, *p* values < 0.001), and correlations among subscales were moderate (factors 1 and 2 = 0.67, factors 1 and 3 = 0.48, factors 2 and 3 = 0.46, *p* values < 0.001).

### Descriptive statistics

The total scale had a mean score of 3.13 with a standard deviation of 1.34 and a median score of 2.96. Average total scores ranged from 1.00 to 6.72. For factor 1 (Perceived and Anticipated Stigma), scores ranged from 1.00 to 6.88, with a mean of 3.15 ± 1.49 and median of 2.97. Factor 2 (Stereotype Application and Self-Devaluation) scores ranged from 1.00 to 7.00, with a mean of 3.17 ± 1.76 and median of 2.75. Factor 3 scores (Stigma Resistance, with reverse-scored items) also ranged from 1.00 to 7.00, with a mean of 2.99 ± 1.35 and median of 3.00.

**Fig. 1:**
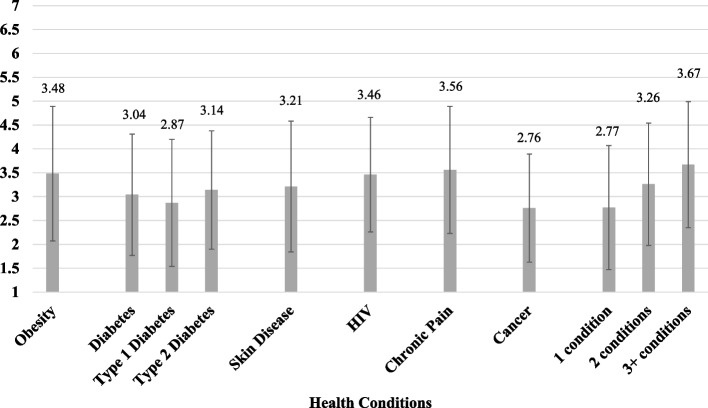
Mean I-HEARTS Scale scores by health condition

Figure 1 displays mean total scores by health condition. When all health condition categories were included in one linear regression model (using square root transformation due to non-normality of I-HEARTS Scale scores), significantly greater scores were found for participants with obesity (*B* = 0.18, *SE* = 0.04, *p* < 0.001), HIV (*B* = 0.19, *SE* = 0.06, *p* = 0.002), and chronic pain (*B* = 0.20, *SE* = 0.04, *p* < 0.001). A one-way nonparametric ANOVA (Kruskal-Wallis test) also showed that scores were higher among participants with more health conditions (*X*^2^[2] = 21.39, *p* < 0.001): Wilcoxon rank sum tests showed that participants who reported three or more of the six types of conditions scored significantly higher than those with one condition (3.67 ± 1.32 vs. 2.77 ± 1.30, *p* < 0.001), though their scores did not significantly differ from those of participants with two types of conditions (3.26 ± 1.28, *p* = 0.121). Participants with two types of health conditions scored significantly higher than those with one condition (*p* = 0.004).

### Associations with demographic characteristics

Spearman’s rank correlations showed that I-HEARTS Scale scores were inversely associated with age (*rho* = −0.27, *p* < 0.001) and years of education (*rho* = −0.33, *p* < 0.001). Kruskal-Wallis tests revealed no significant differences in I-HEARTS Scale scores by participant sex, gender, or race. Hispanic participants scored higher than non-Hispanic participants: 3.58 ± 1.22 vs. 3.06 ± 1.35, *X*^2^(1) = 6.09, *p* = 0.014. Results also revealed significant differences by participant sexual orientation (*X*^2^[3] = 11.29, *p* = 0.010), marital status (*X*^2^[3] = 17.63, *p* < 0.001), and employment status (*X*^2^[5] = 58.48, *p* < 0.001). Pairwise comparisons suggested that scores were higher in bisexual versus heterosexual participants (3.73 ± 1.31 vs. 2.95 ± 1.27, *p* = 0.029); single (3.51 ± 1.44, *p* = 0.002) or divorced participants (3.61 ± 1.50, *p* = 0.023), compared to participants who were married or living with a partner (2.81 ± 1.14); participants who were not working due to disability (4.24 ± 1.23) or were unemployed (4.12 ± 1.16) compared to those who were employed full- (3.02 ± 1.22) or part-time (2.76 ± 1.39), or were retired (2.50 ± 1.12, all *p* values for disability comparisons <0.001; *p* values for unemployed comparisons were 0.002, 0.003, and <0.001, respectively). Scores also differed by income level (*X*^2^[3] = 37.83, *p* < 0.001), with the highest scores among those with incomes <$50,000 (3.67 ± 1.41 vs. 3.02 ± 1.22 for participants with incomes $50,000–99,999, *p* = 0.010, and vs. 2.47 ± 1.07 for incomes of $100,000 or above, *p* < 0.001) and the lowest scores among participants with incomes of $100,000 or above (*p* = 0.019 compared to participants with incomes $50,000–99,999).

### Associations with health condition characteristics

More than two-thirds (69.3%) of the sample endorsed a lifetime experience of being stigmatized by others because of their health conditions. These participants scored significantly higher than those who reported no stigmatizing experiences on the I-HEARTS Scale (3.53 ± 1.26 vs. 2.22 ± 1.03, *X*^2^[1] = 68.95, *p* < 0.001) and all subscales (*p* values < 0.001).

I-HEARTS Scale scores differed significantly depending on the visibility of the participants’ health condition(s): *X*^2^(2) = 15.83, *p* < 0.001. Participants who indicated that they had a health condition that was visible all of the time had the highest scores (3.58 ± 1.36) compared to participants who reported that their condition was visible sometimes (3.05 ± 1.34, *p* = 0.016) or not visible (2.79 ± 1.19, *p* < 0.001). No significant differences were found between participants whose conditions were visible sometimes versus not visible at all. When examining subscales, results were consistent for factors 1 and 2, but no significant differences were found in factor 3 scores depending on visibility of the health condition.

Overall symptom severity was also significantly correlated with I-HEARTS Scale total scores (*rho* = 0.41, *p* < 0.001) and all subscales (*rho* = 0.43, 0.20, and 0.29 for factors 1, 2, and 3, respectively, *p* values < 0.001). In addition, condition-specific symptom severity ratings were significantly correlated with I-HEARTS Scale total scores for all health conditions (*rho* ranged from 0.24 to 0.36, *p* values ranged from 0.015 to <0.001); factor 1 (Perceived and Anticipated Stigma) correlated with condition-specific severity for all health conditions, while factors 2 and 3 (Stereotype Application and Self-Devaluation, and Stigma Resistance) were inconsistently correlated across health conditions.

Duration of health conditions (in months) did not significantly correlate with I-HEARTS Scale total scores for most health conditions, with the exception of HIV (*rho* = −0.45, *p* = 0.001, all subscales also significantly correlated with HIV duration) and cancer (*rho* = −0.23, *p* = 0.046, no subscales correlated with cancer duration). When controlling for the confounding factor of age, no I-HEARTS total scores were significantly associated with condition duration, and only isolated subscale scores were significant (e.g., HIV duration was inversely associated with the Stereotype Application and Self-Devaluation subscale).

### Validity

Supplemental Table S7 presents mean scores on all psychosocial measures. Notably 41.7% of the sample reported PHQ-9 scores in the moderate to severe depression category, and 30.7% reported GAD-7 scores in the moderate to severe anxiety category. Table 5 displays Spearman’s rank correlations between the I-HEARTS Scale total score and each subscale with all psychosocial variables included in the study. Correlations ranged from moderate to strong for the total scale and all subscales. Correlations were stronger for conceptually overlapping variables (e.g., internalized shame, emotional representations of illness) and weaker for measures of related but distinct constructs (physical health-related quality of life, general self-efficacy). The I-HEARTS Scale total score and subscales also significantly correlated with the single-item, 1–5 rating of the extent to which participants’ health conditions affected how they thought and felt about themselves: total score *rho* = 0.63, *p* < 0.001, subscale *rho*s were 0.60, 0.54, and 0.40 for factors 1, 2, and 3, respectively, *p* values < 0.001. Overall, correlations suggested robust associations yet separation between the I-HEARTS Scale and relevant psychosocial variables.

**Table 5:** Correlations of I-HEARTS Scale with psychosocial measures

	Total scale	Factor 1: Perceived and Anticipated Stigma	Factor 2: Stereotype Application and Self-Devaluation	Factor 3: Stigma Resistance
Internalized Shame Scale				
Shame	0.71***	0.68***	0.60***	0.44***
Self-Esteem	−0.62***	−0.55***	−0.51***	−0.63***
UCLA Loneliness Scale	0.65***	0.64***	0.48***	0.45***
IPQ—Emotional	0.73***	0.69***	0.59***	0.53***
PHQ-9	0.58***	0.55***	0.50***	0.42***
GAD-7	0.57***	0.54***	0.49***	0.38***
Social Anxiety Disorder	0.64***	0.62***	0.52***	0.37***
CDC Healthy Days				
Self-rated health	0.45***	0.41***	0.33***	0.45***
Unhealthy days, physical	0.45***	0.45***	0.26***	0.35***
Unhealthy days, mental	0.56***	0.54***	0.44***	0.42***
Total unhealthy days	0.54***	0.53***	0.36***	0.43***
WHODAS 2.0	0.68***	0.69***	0.42***	0.46***
General Self-Efficacy	−0.39***	−0.34***	−0.34***	−0.44***
Perceived Stress Scale	0.61***	0.57***	0.45***	0.51***

Table presents Spearman’s rank correlations. Factor 3 Stigma Resistance items were reverse-scored, such that higher scores indicate greater internalized stigma. *IPQ*, Revised Illness Perceptions Questionnaire (Emotional Representations subscale); *PHQ-9*, Patient Health Questionnaire-9; *GAD-7*, Generalized Anxiety Disorder-7; *CDC*, Centers for Disease Control; for self-rated health, higher scores indicate poorer health; for unhealthy days, higher scores indicate more unhealthy days; *WHODAS*, World Health Organization Disability Assessment Schedule, higher scores indicate more impaired functioning

***p < 0.001

### Cutoff score

K-means clustering and ROC curve analysis were performed to determine a cutoff score using three indices: Youden index, closest to (0,1) index, and Liu’s concordance index. Cutoff scores in reference to the PHQ-9 cutoff score of 10 or above (indicating moderate to severe depression) ranged from 2.88 to 2.90. In reference to the GAD-7 cutoff, scores ranged from 3.20 to 3.78. The cutoff scores by k-means clustering based on all 25 scale items (without reference to an external metric) were 3.44 (Youden index), 3.54 (closest to [0,1] index), and 3.41 (Liu’s concordance index). For k-means clustering based on the factors identified with principal component analysis, cutoff scores were 3.38, 3.38, and 3.41, respectively, for the three indices. Taken together, a final cutoff score of 3.40 was selected to indicate clinically significant internalized health-related stigma. Forty percent of the sample reported this score or higher.

Kruskal-Wallis test results showed significantly higher I-HEARTS Scale total scores among those with moderate to severe depression or anxiety (*X*^2^[1] = 71.55 for PHQ-9 and *X*^2^[1] = 64.09 for GAD-7, *p* values < 0.001), with mean I-HEARTS Scale scores of 3.91 ± 1.30 and 4.07 ± 1.26 among those with PHQ-9 or GAD-7 scores of 10 or above, compared to mean scores of 2.56 ± 1.05 and 2.71 ± 1.14 for those with scores below 10, respectively. Dividing the sample into participants with I-HEARTS Scale scores < vs. ≥3.40, PHQ-9 scores were significantly higher among participants who scored 3.40 or above (13.14 ± 6.56 vs. 6.13 ± 5.31, *X*^2^[1] = 74.22, *p* < 0.001) as were GAD-7 scores (10.78 ± 6.06 vs. 4.79 ± 4.62, *X*^2^[1] = 70.90, *p* < 0.001). Similarly, logistic regression models showed that participants who scored 3.40 or above on the I-HEARTS Scale had significantly higher odds of having moderate to severe depression (OR = 6.00, 95% CI = [3.64, 10.06], *p* < 0.001) and anxiety (OR = 6.70; 95% CI = [3.93, 11.70]; *p* < 0.001). Specifically, of participants who scored ≥3.40, 66.7% reported moderate to severe symptoms of depression, and 54.2% reported moderate to severe anxiety symptoms. Overall, results suggested the cutoff score of 3.40 predicted clinically meaningful levels of depression and anxiety.

## Discussion

This study developed and tested a transdiagnostic measure of internalized health-related stigma in a sample of adults with different kinds of stigmatized chronic physical health conditions. To our knowledge, this was the first stigma measure to be developed with input from community members and health professionals with expertise on a wide range of health conditions that varied in domains relevant to stigma (e.g., visibility, perceived controllability and contagion). It was also the first study to validate an internalized stigma measure with a sample of adults with varied and commonly stigmatized health conditions (obesity, diabetes, HIV, skin diseases, chronic pain, and cancers, among others). As such, this study provides evidence to support use of a single measure of internalized stigma that may be administered in research and clinical care settings for persons with a variety of physical health conditions.

Results showed strong psychometric properties of the 25-item scale and its three factors of Perceived and Anticipated Stigma, Stereotype Application and Self-Devaluation, and Stigma Resistance. These subscales correspond with existing models of internalized stigma that emphasize stigma awareness, self-application of stereotypes, and diminished self-esteem as part of the internalization process [22], with stigma resistance representing the opposite of internalized stigma (and thus a reverse-scored subscale). Total I-HEARTS Scale scores were higher among individuals with obesity, HIV, or chronic pain (in comparison to those with diabetes, skin diseases, or cancers) and among individuals with more than one of the six categories of stigmatized health conditions. Scores were comparable across participant sex, gender, and race, although they were higher among Hispanic compared to non-Hispanic participants. Scores were also higher among younger adults, sexual minority participants, single and divorced individuals, participants who were not working due to disability or were unemployed, and those with lower education and income. Future research with diverse samples will be important for determining whether some groups may be at greater risk for internalizing health-related stigma across conditions.

When examining associations between internalized stigma and disease-related factors, longer duration of disease was negatively associated with I-HEARTS Scale scores for some health conditions (HIV and cancers), although this relationship was largely confounded by age. Other disease-related factors that were associated with higher scores included greater symptom severity and visibility of health conditions. This latter finding is somewhat in contrast to the higher scores observed among participants with HIV and chronic pain (which are often not visible to others); notably, many of these participants also had other health conditions that may have been more visible (e.g., obesity). Compared to concealable health conditions, visible signs of disease may elicit more stigma from others, thus increasing risk for internalizing stigma [31, 74]. Visible symptoms may also increase pressure to disclose or explain one’s health condition (e.g., reassuring others that psoriasis lesions are not contagious), increasing self-consciousness and the possible negative consequences of disclosure (e.g., social exclusion) that compound stigma [75]. In addition, individuals with visible signs of their health conditions may struggle with poor body image or feelings of disgust about their appearance [42], which may also diminish their self-esteem. At the same time, individuals living with concealable health conditions may be vulnerable to internalizing stigma due in part to reduced opportunities for social support (which could buffer against internalization [74]) and heightened distress related to disclosure [76]. Additional research is needed to understand the unique challenges of having a visible versus concealable health condition and its implications for stigma.

I-HEARTS Scale total scores and subscale scores significantly correlated with relevant psychosocial measures of shame, loneliness, stress, and self-efficacy, as well as with depression, anxiety, and HRQOL. Furthermore, a cutoff score of 3.40 on the 1–7 scale predicted clinically meaningful symptoms of depression and anxiety (if scores were summed, this would equate a score of 85 on a scale of 25–175). With further testing and replication (including with confirmatory factor analysis), the I-HEARTS Scale could be utilized in clinical settings to identify patients with high levels of internalized stigma who may benefit from additional psychosocial support. It could also be used in research (especially intervention studies) to recruit individuals who score above the cutoff on the scale, or to further develop a phenotype of individuals with high scores by comparing their characteristics to individuals without clinically significant levels of internalized stigma.

Strengths of the current study include: the extensive measure adaptation process that incorporated perspectives of diverse individuals with different chronic health conditions and health professionals who specialize in these conditions; testing the scale in a large sample of adults with a wide range of health conditions; and comprehensive assessment of psychometrics that yielded strong results. A limitation of the study is that, while representing a broad range of health conditions, the study focused predominantly on six categories (obesity, diabetes, skin disease, HIV, chronic pain, and cancer), so additional research is needed to determine the scale’s psychometrics and validity in other populations. The new measure was adapted from items that were initially developed to assess stigma due to mental illness; although these items had been previously used in samples with stigmatized physical health conditions [47] and were thoroughly reviewed and modified according to feedback from Advisory Board members, item content may have differed if developed solely for the purpose of capturing stigma concerns related to physical health. The I-HEARTS Scale’s three-factor structure differed from the original ISMI Scale’s five-factor structure; this may be due in part to differences in physical versus mental health stigma (as reflected by the changes made to the original scale items), although other studies of mental health stigma have also found alternative factor structures [47]. Replication of the findings with confirmatory factor analysis is needed, especially in a sample with greater diversity of gender, race, ethnicity, and education, and perhaps among individuals with mental health conditions as well. Although 2-week test-retest reliability was assessed in the current study, testing the measure’s reliability over a longer period of time and its responsiveness and sensitivity to change will further contribute to knowledge of its utility in longitudinal and intervention research. Finally, although multiple steps were taken to ensure data quality and integrity from this online sample, replication of this research in a sample of patients who are enrolled through clinics (with confirmed diagnoses) would reinforce confidence in the measure’s psychometric properties.

## Conclusions

The current study developed and tested a transdiagnostic measure of internalized health-related stigma that can be disseminated to adults with different kinds of physical health conditions. Results showed that the I-HEARTS Scale had strong psychometric properties, and a cutoff score was able to determine clinically meaningful profiles of depression and anxiety. Findings also revealed some differences in internalized stigma across characteristics of participants and their health conditions that warrant further exploration. This research aids efforts to capture experiences of adults with multiple comorbid health conditions (reflecting real-world clinical presentations) in order to identify individuals who may be at the highest risk for internalizing stigma. This measure also has the potential to bridge knowledge across health fields by allowing for comparisons of stigma across different types of health conditions and by facilitating research that is less siloed in its approach to addressing internalized stigma (e.g., by including participants with different kinds of health conditions within the same intervention studies). With further replication of its reliability and validity, the I-HEARTS Scale may be used in cross-disciplinary research and in clinical settings to advance understanding of and interventions for internalized health-related stigma.

## Supplementary Information

Additional file 1: Supplemental Table S1. Demographic characteristics of Advisory Board members. Supplemental Table S2. Advisory Board ratings of initial scale items, *n* (%). Supplemental Table S3. Additional details of health conditions reported in the categories of skin diseases, chronic pain, and cancers. Supplemental Table S4. Factor analysis of 30-item I-HEARTS Scale. Supplemental Table S5. Confirmatory factor analyses to test for measurement invariance across participants with one or multiple health conditions and across health conditions categories. Supplemental Table S6. Item-level item-total correlations for 25-item I-HEARTS Scale. Supplemental Table S7. Mean scores on psychosocial measures (*N* = 300).

## Data Availability

Data and/or research tools used in the preparation of this manuscript were obtained from the National Institute of Mental Health (NIMH) Data Archive (NDA). NDA is a collaborative informatics system created by the National Institutes of Health to provide a national resource to support and accelerate research in mental health. Dataset identifier(s): doi: 10.15154/6zmg-zs57 [77]. This manuscript reflects the views of the authors and may not reflect the opinions or views of the NIH or of the submitters submitting original data to NDA.

## Abbreviations

ANOVA: Analysis of variance
GAD-7: Generalized Anxiety Disorder-7
HRQOL: Health-related quality of life
I-HEARTS Scale: Internalized Health-Related Stigma Scale
ISMI Scale: Internalized Stigma of Mental Illness Scale
PHQ-9: Patient Health Questionnaire-9
WHODAS: World Health Organization Disability Assessment Schedule

## References

[1] Stangl AL, Earnshaw VA, Logie CH, et al. The health stigma and discrimination framework: a global, crosscutting framework to inform research, intervention development, and policy on health-related stigmas. BMC Med. 2019;17:31. 30764826 10.1186/s12916-019-1271-3 PMC6376797

[2] Brewis A, SturtzSreetharan C, Wutich A. Obesity stigma as a globalizing health challenge. Glob Health. 2018;14:20. 10.1186/s12992-018-0337-x. 10.1186/s12992-018-0337-x PMC5811962 29439728

[3] Liu NF, Brown AS, Folias AE, et al. Stigma in people with type 1 and type 2 diabetes. Clin Diabetes. 2017;35(1):27–34. 28144043 10.2337/cd16-0020 PMC5241772

[4] Topp J, Andrees V, Weinberger NA, et al. Strategies to reduce stigma related to visible chronic skin diseases: a systematic review. J Eur Acad Dermatol Venereol. 2019;33:2029–38. 31177601 10.1111/jdv.15734

[5] Pearl RL, Wan MT, Takeshita J, Gelfand JM. Stigmatizing attitudes toward persons wtih psoriasis among laypersons and medical students. J Am Acad Dermatol. 2019;80(6):1556–63. 30171876 10.1016/j.jaad.2018.08.014 PMC6688164

[6] Earnshaw VA, Smith LR, Chaudoir SR, Amico KR, Copenhaver MM. HIV stigma mechanisms and well-being among PLWH: a test of the HIV stigma framework. AIDS Behav. 2013;17(5):1785–95. 23456594 10.1007/s10461-013-0437-9 PMC3664141

[7] Carr DB. President’s message: patients with pain need less stigma, not more. Pain Med. 2016;17:1391–3. 27418318 10.1093/pm/pnw158

[8] Fujisawa D, Hagiwara N. Cancer stigma and its health consequences. Curr Breast Cancer Rep. 2015;7:143–250.

[9] Scambler G. Health-related stigma. Sociol Health Illn. 2009;31(3):441–55. 19366430 10.1111/j.1467-9566.2009.01161.x

[10] Weiss MG, Ramakrishna J, Somma D. Health-related stigma: rethinking concepts and interventions. Psychol Health Med. 2006;11(3):277–87. 17130065 10.1080/13548500600595053

[11] van Brakel WH. Measuring health-related stigma: a literature review. Psychol Health Med. 2006;11(3):307–34. 17130068 10.1080/13548500600595160

[12] van Brakel WH, Cataldo J, Grover S, et al. Out of the silos: identifying cross-cutting features of health-related stigma to advance measurement and intervention. BMC Med. 2019;17:13. 10.1186/s12916-018-1245-x. 30764817 10.1186/s12916-018-1245-x PMC6376667

[13] Hatzenbuehler ML, Phelan JC, Link BG. Stigma as a fundamental cause of population health disparities. Am J Public Health. 2013;103(5):813–21. 23488505 10.2105/AJPH.2012.301069 PMC3682466

[14] Pachankis JE, Hatzenbuehler ML, Wang K, et al. The burden of stigma on health and well-being: a taxonomy of concealment, course, disruptiveness, aesthetics, origin, and peril across 93 stigmas. Pers Soc Psychol Bull. 2018;44(4):451–74. 29290150 10.1177/0146167217741313 PMC5837924

[15] Ebuenyi I, Taylor C, O’Flynn D, Matthew Prina A, Passchier R, Mayston R. The impact of co-morbid severe mental illness and HIV upon mental and physical health and social outcomes: a systematic review. AIDS Care. 2018;30(12):1586–94. 30114950 10.1080/09540121.2018.1510110

[16] Avila C, Holloway AC, Hahn MK, et al. An overview of links between obesity and mental health. Curr Obes Rep. 2015;4:303–10. 26627487 10.1007/s13679-015-0164-9

[17] Cortes H, Rojas-Marquez M, Del Prado-Audelo ML, Reyes-Hernandez OD, Gonzalez-Del Carmen M, Leyva-Gomez G. Alterations in mental health and quality of life in patients with skin disorders: a narrative review. Int J Dermatol. 2022;61(7):783–91. 34403497 10.1111/ijd.15852

[18] Singer S, Das-Munshi J, Bruhler E. Prevalence of mental health conditions in cancer patients in acute care: a meta-analysis. Ann Oncol. 2010;21(5):925–30. 19887467 10.1093/annonc/mdp515

[19] Hooten WM. Chronic pain and mental health disorders: shared neural mechanisms, epidemiology, and treatment. Mayo Clin Proc. 2016;91(7):955–70. 27344405 10.1016/j.mayocp.2016.04.029

[20] Diabetes Canada Clinical Practice Guidelines Expert Committee, Robinson DJ, Coons M, Haensel H, Vallis M, Yale JF. Diabetes and mental health. Can J Diabetes. 2018;41(S1):S130–41. 10.1016/j.jcjd.2017.10.031 29650085

[21] Ma PHX, Chan XCY, Loke AY. Self-stigma reduction interventions for people living with HIV/AIDS and their families: a systematic review. AIDS Behav. 2019;23:707–41. 30298241 10.1007/s10461-018-2304-1

[22] Corrigan PW, Rao D. On the self-stigma of mental illness: stages, disclosure, and strategies for change. Can J Psychiatry. 2012;57(8):464–9. 22854028 10.1177/070674371205700804 PMC3610943

[23] Alpsoy E, Polat M, Fettahliouglu-Karaman B, et al. Internalized stigma in psoriasis: a multicenter study. J Dermatol. 2017;44(8):885–91. 28407292 10.1111/1346-8138.13841

[24] Durso LE, Latner JD. Understanding self-directed stigma: development of the weight bias internalization scale. Obesity. 2008;16:S80–6. 18978768 10.1038/oby.2008.448

[25] Browne JL, Ventura AD, Mosely K, Speight J. Measuring the stigma surrounding type 2 diabetes: development and validation of the Type 2 Diabetes Stigma Assessment Scale (DSAS-2). Diabetes Care. 2016;39:2141–8. 27515964 10.2337/dc16-0117

[26] Fife BL, Wright ER. The dimensionality of stigma: a comparison to its impact on the self of persons with HIV/AIDS and cancer. J Health Soc Behav. 2000;41(1):50–67. 10750322

[27] Stevelink SAM, Wu IC, Voorend CGN, van Brakel WH. The psychometric assessment of internalized stigma instruments: a systematic review. Stigma Res Action. 2012;2(2):100–18.

[28] Waugh OC, Byrne DG, Nicholas MK. Internalized stigma in people living with chronic pain. J Pain. 2014;15(5):550.e1-550.e10. 24548852 10.1016/j.jpain.2014.02.001

[29] Kato A, Fujimaki Y, Fujimori S, et al. A qualitative study on the impact of internalized stigma on type 2 diabetes self-management. Patient Educ Couns. 2016;99:1233–9. 27873575 10.1016/j.pec.2016.02.002

[30] Germain N, Augustin M, Francois C, et al. Stigma in visible skin diseases: a literature review and development of a conceptual model. J Eur Acad Dermatol Venereol. 2021;35(7):1493–504. 33428316 10.1111/jdv.17110

[31] Romano KA, Heron KE, Sandoval CM, Howard LM, MacIntyre RI, Mason TB. A meta-analysis of associations between weight bias internalization and conceptually-related correlates: a step toward improving construct validity. Clin Psychol Rev. 2022;92:102127. 35074712 10.1016/j.cpr.2022.102127 PMC8858873

[32] Corrigan PW, Watson AC, Barr L. The self-stigma of mental illness: implications for self-esteem and self-efficacy. J Soc Clin Psychol. 2006;25(9):875–84.

[33] Pearl RL, Puhl RM, Himmelstein MS, Pinto AM, Foster GD. Weight stigma and weight-related health: associations of self-report measures among adults in weight management. Ann Behav Med. 2020;54(11):904–14. 32333673 10.1093/abm/kaaa026 PMC7646152

[34] Goffman E. Stigma: notes on the management of a spoiled identity. Englewood Cliffs: Prentice Hall; 1963.

[35] van der Kooij YL, den Daas C, Bos AER, Willems RA, Stutterheim SE. Correlates of internalized HIV stigma: a comprehensive systematic review. AIDS Educ Prev. 2023;35(2):158–72. 37129595 10.1521/aeap.2023.35.2.158

[36] Else-Quest NM, Jackson TL. Cancer stigma. In: Corrigan P, editor. The stigma of disease and disability: understanding causes and overcoming injustices. Washington, DC: American Psychological Association; 2014. p. 165–81.

[37] Ramos Salas X, Forhan M, Caulfield T, Sharma AM, Raine KD. Addressing internalized weight bias and changing damaged social identities for people living with obesity. Front Psychol. 2019;10. 10.3389/fpsyg.2019.01409. 10.3389/fpsyg.2019.01409 PMC6606721 31293476

[38] Braun TD, Gorin AA, Puhl RM, et al. Shame and self-compassion as risk and protective mechanisms of the internalized weight bias and emotional eating link in individuals seeking bariatric surgery. Obes Surg. 2021;31:3177–87. 33905070 10.1007/s11695-021-05392-z PMC8493808

[39] Turan B, Smith W, Cohen MH, et al. Mechanisms for the negative effects of internalized HIV-related stigma on ART adherence in women: the mediating roles of social isolation and depression. J Acquir Immune Defic Syndr. 2016;72(2):198–205. 26885803 10.1097/QAI.0000000000000948 PMC4868649

[40] Bean DJ, Dryland A, Rashid U, Tuck NL. The determinants and effects of chronic pain stigma: a mixed methods study and the development of a model. J Pain. 2022;23(10):1749–64. 35700874 10.1016/j.jpain.2022.05.006

[41] Browne JL, Ventura AD, Mosely K, Speight J. Measuring type 1 diabetes stigma: development and validation of the type 1 Diabetes Stigma Assessment Scale (DSAS-1). Diabet Med. 2017;34(12):1773–82. 28891210 10.1111/dme.13507

[42] Pearl RL, Puhl RM. Weight bias internalization and health: a systematic review. Obes Rev. 2018;19(8):1141–63. 29788533 10.1111/obr.12701 PMC6103811

[43] Puhl RM, Himmelstein MS, Hateley-Browne JL, Speight J. Weight stigma and diabetes stigma in U.S. adults with type 2 diabetes: associations with diabetes self-care behaviors and perceptions of health care. Diabetes Res Clin Pract. 2020;168:108387. 32858100 10.1016/j.diabres.2020.108387

[44] Scott W, Yu L, Patel S, McCracken LM. Measuring stigma in chronic pain: preliminary investigation of instrument psychometrics, correlates, and magnitude of change in a prospective cohort attending interdisciplinary treatment. J Pain. 2019;20(10):1164–75. 30940501 10.1016/j.jpain.2019.03.011

[45] Luck-Sikorski C, Robmann P, Topp J, Augustin M, Sommer R, Weinberger NA. Assessment of stigma related to visible skin diseases: a systematic review and evaluation of patient-reported outcomes measures. Jounral Eur Acad Dermatol Venereol. 2022;36(4):499–525. 10.1111/jdv.17833 34817889

[46] Boersma P, Black LI, Ward BW. Prevalence of multiple chronic conditions among US adults, 2018. Prev Chronic Dis. 2020;17:E106. 10.5888/pcd17.200130. 32945769 10.5888/pcd17.200130 PMC7553211

[47] Boyd JE, Adler EP, Otilingam PG, Peters T. Internalized Stigma of Mental Illness (ISMI) Scale: a multinational review. Compr Psychiatry. 2014;55:221–31. 24060237 10.1016/j.comppsych.2013.06.005

[48] Terwee CB, Prinsen C, Chiarotto A, et al. COSMIN methodology for assessing the content validity of PROMs: user manual. Amsterdam: VU University Medical Center; 2018.

[49] Boyd Ritsher J, Otilingam PG, Grajales M. Internalized stigma of mental illness: psychometric properties of a new measure. Psychiatry Res. 2003;121(1):31–49. 14572622 10.1016/j.psychres.2003.08.008

[50] Rensen C, Bandyopadhyay S, Gopal PK, Van Brakel WH. Measuring leprosy-related stigma: a pilot study to validate a toolkit of instruments. Disabil Rehabil. 2011;33(9):711–9. 20690861 10.3109/09638288.2010.506942

[51] Alpsoy E, Senol Y, Temel AB, Baysal GO, Karakas AA. Reliability and validity of internalized stigmatization scale in psoriasis. Turk Arch Dermatol Adn Venereol. 2015;49(1):45–9.

[52] Stevelink SAM, van Brakel WH, Augustine V. Stigma and social participation in Southern India: differences and commonalities among persons affected by leprosy and persons living with HIV/AIDS. Psychol Health Med. 2011;16(6):695–707. 21391136 10.1080/13548506.2011.555945

[53] Cook DR. Measuring shame: the Internalized Shame Scale. Alcohol Treat Q. 1987;4:197–215.

[54] Rybak CJ, Brown B. Assessment of internalized shame: validity and reliability of the internalized shame scale. Alcohol Treat Q. 1996;14(1):71–83.

[55] Del Rosario PM, White RM. The Internalized Shame Scale: temporal stability, internal consistency, and principal components analysis. Personal Individ Differ. 2006;41(1):95–103.

[56] Russell DW. UCLA Loneliness Scale (version 3): reliability, validity, and factor structure. J Pers Assess. 1996;66(2):20–40. 8576833 10.1207/s15327752jpa6601_2

[57] Moss-Morris R, Weinman J, Petrie K, Horne R, Cameron L, Buick D. The revised illness perception questionnaire (IPQ-R). Psychol Health. 2002;17(1):1–16.

[58] Schwarzer R, Jerusalem M. Generalized self-efficacy scale. In: Measures in health psychology: a user’s portfolio. causal and control beliefs. Windsor: NFER-NELSON; 1995. p. 35–7.

[59] Cohen S, Kamarck T, Mermelstein R. A global measure of perceived stress. J Health Soc Behav. 1983;24(4):385–96. 6668417

[60] Lee EH. Review of the psychometric evidence of the perceived stress scale. Asian Nurs Res. 2012;6(4):121–7. 10.1016/j.anr.2012.08.004 25031113

[61] Kroenke K, Spitzer RL, Williams JBW. The PHQ-9: validity of a brief depression severity measure. J Gen Intern Med. 2001;16(9):600–13. 10.1046/j.1525-1497.2001.016009606.x PMC1495268 11556941

[62] Spitzer RL, Kroenke K, Williams JB, Lowe B. A brief measure assessing generalized anxiety disorder: the GAD-7. Arch Intern Med. 2006;166(10):1092–7. 16717171 10.1001/archinte.166.10.1092

[63] Craske M, Wittchen U, Bogels S, Stein M, Andrews G, Lebeu R. Severity measure for social anxiety disorder (social phobia) - adult. https://www.psychiatry.org/psychiatrists/practice/dsm/educational-resources/assessment-measures. Published online 2013.

[64] Moriarty DG, Zack MM, Kobau R. The centers for disease control and prevention’s healthy days measures – population tracking of perceived physical and mental health over time. Health Qual Life Outcomes. 2003;1(37):1–8. 14498988 10.1186/1477-7525-1-37 PMC201011

[65] Ustun TB, Kostanjsek N, Chatterji S, Rehm J. Measuring health and disability: manual for WHO Disability Assessment Schedule WHODAS 2.0. World Health Organization; 2010. https://iris.who.int/bitstream/handle/10665/43974/9789241547598_eng.pdf?sequence=1.

[66] Watson PF, Petrie A. Method agreement analysis: a review of correct methodology. Theriogenology. 2010;73:1167–79. 20138353 10.1016/j.theriogenology.2010.01.003

[67] Bujang MA, Omar ED, Foo DHP, Hon YK. Sample size determination for conducting a pilot study to assess reliability of a questionnaire. Restor Dent Endod. 2024;49(1):e3. 38449496 10.5395/rde.2024.49.e3 PMC10912549

[68] Plummer F, Manea L, Trepel D, McMillan D. Screening for anxiety disorders with the GAD-7 and GAD-2: a systematic review and diagnostic metaanalysis. Gen Hosp Psychiatry. 2016;39:24–31. 26719105 10.1016/j.genhosppsych.2015.11.005

[69] Costantini L, Pasquarella C, Odone A, et al. Screening for depression in primary care with Patient Health Questionnaire-9 (PHQ-9): a systematic review. J Affect Disord. 2021;279:473–83. 33126078 10.1016/j.jad.2020.09.131

[70] American Psychiatric Association. DSM-5TR-online assessment measures. https://www.psychiatry.org/psychiatrists/practice/dsm/educational-resources/assessment-measures.

[71] Acar Guvendir M, Ozer OY. Item removal strategies conducted in exploratory factor analysis: a comparative study. Int J Assess Tools Educ. 2022;9:165–80.

[72] Mirabelli J, Jensen KA, Vohra SR, Johnson E. Exploring the exploratory factor analysis: comparisons and insights from applying five procedures to determining EFA item retention. In: American Society for Engineering Education. 2022. Paper ID# 37265.

[73] Costello A, Osborne JW. Best practices in exploratory factor analysis: four recommendations for getting the most from your analysis. Pract Assess Res Eval. 2005;10:1–9.

[74] Fazeli PL, Turan JM, Budhwani H, et al. Moment-to-moment within-person associations between acts of discrimination and internalized stigma in people living with HIV: an experience sampling study. Stigma Health. 2017;2(3):216–28. 28966982 10.1037/sah0000051 PMC5614514

[75] Corrigan PW, Kosyluk K, Rusch N. Reducing self-stigma by coming out proud. Am J Public Health. 2013;103:794–800. 23488488 10.2105/AJPH.2012.301037 PMC3698841

[76] Chaudoir SR, Earnshaw VA, Andel S. “Discredited” versus “discreditable”: understanding how shared and unique stigma mechanisms affect psychological and physical health disparities. Basic Appl Soc Psychol. 2013;35(1):75–87. 10.1080/01973533.2012.746612 PMC3666955 23729948

[77] Pearl R, Li Y, Groshon L, et al. Measuring internalized health-related stigma across health conditions: development and validation of the I-HEARTS Scale. NDA. 10.15154/6zmg-zs57. 10.1186/s12916-024-03661-z PMC11463042 39379928

