# Supplementary material for: Measuring internalized health-related stigma across health conditions: development and validation of the I-HEARTS Scale
Source: BMC Med. 2024 Oct 8;22:435. doi: 10.1186/s12916-024-03661-z (PMC11463042; doi:10.1186/s12916-024-03661-z)
Supplement: Supplementary file 1 — Additional file 1: Supplemental Table S1. Demographic characteristics of Advisory Board members. Supplemental Table S2. Advisory Board ratings of initial scale items, n (%). Supplemental Table S3. Additional details of health conditions reported in the categories of skin diseases, chronic pain, and cancers. Supplemental Table S4. Factor analysis of 30-item I-HEARTS Scale. Supplemental Table S5. Confirmatory factor analyses to test for measurement invariance across participants with one or multiple health conditions and across health conditions categories. Supplemental Table S6. Item-level item-total correlations for 25-item I-HEARTS Scale. Supplemental Table S7. Mean scores on psychosocial measures (N = 300). [file 12916_2024_3661_MOESM1_ESM.docx]

Supplemental Table S1. Demographic characteristics of Advisory Board members

| Variable | Health Professionals (*N*=11) | Community Members (*N*=12) |
| --- | --- | --- |
|  | *Mean*±*SD* or *n* | *Mean*±*SD* or *n* |
| Age (years) | 53.2±13.2 | 49.6±11.4 |
| Gender |  |  |
| Male | 4 | 3 |
| Female | 7 | 8 |
| Non-binary/third gender | 0 | 1 |
| Race |  |  |
| Asian | 2 | 1 |
| Black or African American | 0 | 4 |
| White | 9 | 6 |
| Other | 0 | 1 |
| Ethnicity |  |  |
| Hispanic or Latino/a/x | 1 | 2 |
| Not Hispanic or Latino/a/x | 9 | 9 |
| Not reported | 1 | 1 |
| Education |  |  |
| Some college | 0 | 2 |
| 4 year degree | 0 | 3 |
| Professional degree | 1 | 4 |
| Doctorate | 10 | 2 |
| Not reported | 0 | 1 |

Note. SD=Standard Deviation

Supplemental Table S2. Advisory Board ratings of initial scale items, *n* (%)

Rating Scale: Relevance/Clarity: 0=no relevance/clarity, 1=somewhat relevant/clear, 2=very relevant/clear

Conclusion: 0=reject, 1=accept with modification, 2=accept

|  | **Relevance** | | | **Total** | **Clarity** | | | **Total** | **Conclusion** | | | **Total** |
| --- | --- | --- | --- | --- | --- | --- | --- | --- | --- | --- | --- | --- |
| **Item** | 0 | 1 | 2 | **N** | 0 | 1 | 2 | **N** | 0 | 1 | 2 | **N** |
| 1. I feel out of place in the world because of my health condition(s)* | 1  (5.3%) | 4 (21.1%) | 14 (73.7%) | 19 | 1  (5.0%) | 8 (40.0%) | 11 (55.0%) | 20 | 2 (10.0%) | 5 (25.0%) | 13 (65.0%) | 20 |
| **2. Having this health condition(s) has ruined my life** | 2 (10.0%) | 3 (15.0%) | 15 (75.0%) | 20 | 0 | 3 (14.3%) | 18 (85.7%) | 21 | 2  (9.5%) | 1  (4.8%) | 18 (85.7%) | 21 |
| **3. People without my health condition(s) could not possibly understand me** | 0 | 4 (20.0%) | 16 (80.0%) | 20 | 0 | 4 (19.1%) | 17 (81.0%) | 21 | 0 | 4 (19.1%) | 17 (81.0%) | 21 |
| **4. I am embarrassed or ashamed that I have this health condition(s)** | 0 | 3 (15.0%) | 17 (85.0%) | 20 | 0 | 3 (14.3%) | 18 (85.7%) | 21 | 0 | 2  (9.5%) | 19 (90.5%) | 21 |
| **5. I am disappointed in myself for having this health condition(s)** | 1  (5.0%) | 7 (35.0%) | 12 (60.0%) | 20 | 1  (4.8%) | 3 (14.3%) | 17 (81.0%) | 21 | 1  (4.8%) | 3 (14.3%) | 17 (81.0%) | 21 |
| **6. I feel that I am a lesser person compared to others who don’t have this health condition(s)** | 2  (9.5%) | 4 (19.1%) | 15 (71.4%) | 21 | 1  (4.8%) | 5 (23.8%) | 15 (71.4%) | 21 | 1  (4.8%) | 4 (19.1%) | 16 (76.2%) | 21 |
| 7. Negative assumptions about people with my health condition(s) apply to me* | 3 (14.3%) | 3 (14.3%) | 15 (71.4%) | 21 | 3 (14.3%) | 7 (33.3%) | 11 (52.4%) | 21 | 3 (14.3%) | 4 (19.1%) | 14 (66.7%) | 21 |
| 8. People with my health condition(s) cannot live a good, rewarding life* | 2  (9.5%) | 3 (14.3%) | 16 (76.2%) | 21 | 1  (4.8%) | 4 (19.1%) | 16 (76.2%) | 21 | 2  (9.5%) | 2  (9.5%) | 17 (81.0%) | 21 |
| 9. I can’t contribute anything to society because I have this health condition(s)* | 3 (14.3%) | 3 (14.3%) | 15 (71.4%) | 21 | 1  (4.8%) | 3 (14.3%) | 17 (81.0%) | 21 | 3 (14.3%) | 2  (9.5%) | 16 (76.2%) | 21 |
| 10. I’m not attractive as a romantic partner because of my health condition(s)* | 0 | 2 (9.5%) | 19 (90.5%) | 21 | 0 | 5 (23.8%) | 16 (76.2%) | 21 | 0 | 2  (9.5%) | 19 (90.5%) | 21 |
| **11. People treat me unfairly because I have this health condition(s)** | 2  (9.5%) | 4 (19.1%) | 15 (71.4%) | 21 | 3 (14.3%) | 5 (23.8%) | 13 (61.9%) | 21 | 2  (9.5%) | 2  (9.5%) | 17 (81.0%) | 21 |
| 12. Others think that I can’t achieve much in life because I have this health condition(s)* | 1  (4.8%) | 7 (33.3%) | 13 (61.9%) | 21 | 1  (4.8%) | 7 (33.3%) | 13 (61.9%) | 21 | 1  (4.8%) | 7 (33.3%) | 13 (61.9%) | 21 |
| 13. People ignore me or take me less seriously just because I have this health condition(s)* | 2  (9.5%) | 4 (19.1%) | 15 (71.4%) | 21 | 2  (9.5%) | 7 (33.3%) | 12 (57.1%) | 21 | 2 (10.0%) | 5 (25.0%) | 13 (65.0%) | 20 |
| *14. People often treat me like a child just because I have this health condition(s)*** | 8 (38.1%) | 5 (23.8%) | 8 (38.1%) | 21 | 7 (33.3%) | 5 (23.8%) | 9 (42.9%) | 21 | 7 (33.3%) | 6 (28.6%) | 8 (38.1%) | 21 |
| 15. Nobody would be interested in getting close to me because I have this health condition(s)* | 3 (14.3%) | 3 (14.3%) | 15 (71.4%) | 21 | 3 (14.3%) | 7 (33.3%) | 11 (52.4%) | 21 | 3 (14.3%) | 5 (23.8%) | 13 (61.9%) | 21 |
| 16. Employers would be less likely to hire me for certain jobs, compared to others who don’t have this health condition(s)* | 2  (9.5%) | 5 (23.8%) | 14 (66.7%) | 21 | 3 (14.3%) | 4 (19.1%) | 14 (66.7%) | 21 | 2  (9.5%) | 4 (19.1%) | 15 (71.4%) | 21 |
| 17. I don’t talk about myself much because I don’t want to burden others with my health condition(s)* | 1  (4.8%) | 6 (28.6%) | 14 (66.7%) | 21 | 0 | 9 (42.9%) | 12 (57.1%) | 21 | 0 | 8 (38.1%) | 13 (61.9%) | 21 |
| 18. I don’t socialize much because I worry what others will think of me due to my health condition(s)* | 3 (14.3%) | 3 (14.3%) | 15 (71.4%) | 21 | 2  (9.5%) | 5 (23.8%) | 14 (66.7%) | 21 | 3 (14.3%) | 4 (19.1%) | 14 (66.7%) | 21 |
| **19. Negative assumptions about people with my health condition(s) keep me isolated from the world around me** | 3 (14.3%) | 4 (19.1%) | 14 (66.7%) | 21 | 1  (4.8%) | 6 (28.6%) | 14 (66.7%) | 21 | 2  (9.5%) | 5 (23.8%) | 14 (66.7%) | 21 |
| 20. I stay away from social situations in order to protect my family or friends from embarrassment* | 3 (15.0%) | 7 (35.0%) | 10 (50.0%) | 20 | 4 (19.1%) | 8 (38.1%) | 9 (42.9%) | 21 | 4 (19.1%) | 8 (38.1%) | 9 (42.9%) | 21 |
| 21. Being around people who don’t have this health condition(s) makes me feel out of place or not good enough* | 1  (4.8%) | 4 (19.1%) | 16 (76.2%) | 21 | 0 | 7 (33.3%) | 14 (66.7%) | 21 | 1  (4.8%) | 5 (23.8%) | 15 (71.4%) | 21 |
| 22. I avoid getting close to people who don’t have this health condition(s) to avoid rejection* | 3 (15.0%) | 2 (10.0%) | 15 (75.0%) | 20 | 2 (10.0%) | 4 (20.0%) | 14 (70.0%) | 20 | 3 (15.0%) | 3 (15.0%) | 14 (70.0%) | 20 |
| 23. I feel comfortable being seen in public with a person who has this health condition(s)* | 1  (4.8%) | 0 (0.0%) | 20 (95.2%) | 21 | 1  (4.8%) | 3 (14.3%) | 17 (81.0%) | 21 | 2 (9.5%) | 3 (14.3%) | 16 (76.2%) | 21 |
| 24. In general, I am able to live life the way I want to* | 0 | 1 (4.8%) | 20 (95.2%) | 21 | 0 | 2 (9.5%) | 19 (90.5%) | 21 | 0 (0.0%) | 1 (4.8%) | 20 (95.2%) | 21 |
| **25. I can have a good, fulfilling life, despite my health condition(s)** | 0 | 1  (4.8%) | 20 (95.2%) | 21 | 0 | 0 (0.0%) | 21 (100.0%) | 21 | 0 (0.0%) | 1 (5.0%) | 19 (95.0%) | 20 |
| **26. People with this health condition(s) make important contributions to society** | 0 | 0 | 21 (100.0%) | 21 | 0 | 1 (5.0%) | 19 (95.0%) | 20 | 0 (0.0%) | 1 (5.0%) | 19 (95.0%) | 20 |
| **27. Living with this health condition(s) has made me a strong person** | 0 | 2 (10.0%) | 18 (90.0%) | 20 | 0 | 3 (15.0%) | 17 (85.0%) | 20 | 0 (0.0%) | 3 (15.0%) | 17 (85.0%) | 20 |

Note. Bolded items were retained in their current form. One item (in italics) was removed. *Items marked with an asterisk were subsequently modified. Based on Advisory Board feedback, three new items were added, and one item (#13) was divided into two items, for a total of 30 items included in the scale. The modified and added items were distributed to Advisory Board members for feedback before finalizing the scale.

Supplemental Table S3. Additional details of health conditions reported in the categories of skin diseases, chronic pain, and cancers

| **Health Conditions** | **N (%)** |
| --- | --- |
|  |  |
| *Specific diagnoses reported for skin diseases* | *n=90* |
| Acne | 3 (3.3%) |
| Alopecia | 4 (4.4%) |
| Eczema | 36 (40.0%) |
| Psoriasis/psoriatic arthritis | 34 (37.8%) |
| Rosacea | 12 (13.3%) |
| Skin cancers | 3 (3.3%) |
| Vitiligo | 6 (6.7%) |
| Other | 10 (11.1%) |
|  |  |
| *Specific locations/diagnoses reported for chronic pain* | *n=113* |
| Arthritis* | 27 (23.9%) |
| Back/Spine | 68 (60.2%) |
| Hip | 13 (11.5%) |
| Knee | 27 (23.9%) |
| Other joints | 14 (12.4%) |
| Shoulder | 11 (9.7%) |
| Head/Migraine | 8 (7.1%) |
| Hands | 13 (11.5%) |
| Feet | 19 (16.8%) |
| Leg | 13 (11.5%) |
| Neuropathy* | 7 (6.2%) |
| Whole body/Fibromyalgia | 15 (13.3%) |
| Other | 15 (13.3%) |
|  |  |
| *Specific types of cancers* | *n=74* |
| Breast | 32 (43.2%) |
| Bladder | 5 (6.8%) |
| Colon/Colorectal | 10 (13.5%) |
| Head/Neck | 5 (6.8%) |
| Lung | 2 (2.7%) |
| Prostate | 12 (16.2%) |
| Female Reproductive | 5 (6.8%) |
| Skin | 8 (10.8%) |
| Thyroid | 4 (5.4%) |
| Other | 4 (5.4%) |
| *Cancer status* |  |
| Active | 10 (13.5%) |
| Remission | 54 (73.0%) |
| Other | 10 (13.5%) |
| Undergoing chemotherapy or radiation | 6 (8.1%) |

Note. Percentages are provided based on the number of participants endorsing each category of health condition. *Many participants who endorsed arthritis also reported specific locations of pain that are included in other codings. Of those who endorsed arthritis, 12 specified osteoarthritis, 8 specified psoriatic arthritis, and 3 specified rheumatoid arthritis. In addition, 4 participants with neuropathy specified that it affected their feet and/or hands, and 1 participant specified that it affected their legs (these participants are also included in location-specific codings). Some participants with skin cancer endorsed a diagnosis of a skin disease, while others endorsed a diagnosis of cancer; as a result, skin cancer is listed twice in the table.

Supplemental Table S4. Factor analysis of 30-item I-HEARTS Scale

| Item | Factor 1 | Factor 2 | Factor 3 |
| --- | --- | --- | --- |
| 1 | **0.69** | 0.17 | 0.08 |
| 2 | **0.69** | 0.05 | 0.18 |
| 3 | **0.85** | -0.18 | -0.04 |
| 4 | 0.28 | **0.69** | 0.04 |
| 5 | 0.11 | **0.85** | 0.01 |
| 6 | -0.10 | **0.93** | 0.01 |
| 7 | 0.47 | 0.43 | 0.11 |
| 8 | 0.30 | **0.52** | 0.05 |
| 9 | 0.53 | 0.06 | 0.43 |
| 10 | **0.66** | -0.13 | 0.34 |
| 11 | 0.40 | 0.32 | 0.19 |
| 12 | **0.84** | 0.10 | -0.13 |
| 13 | **0.77** | 0.02 | 0.02 |
| 14 | **0.70** | 0.22 | -0.04 |
| 15 | **0.88** | 0.03 | -0.08 |
| 16 | **0.87** | -0.13 | -0.11 |
| 17 | **0.74** | 0.23 | -0.02 |
| 18 | **0.85** | -0.15 | 0.02 |
| 19 | **0.67** | 0.16 | -0.14 |
| 20 | **0.77** | 0.15 | 0.07 |
| 21 | **0.75** | 0.23 | 0.01 |
| 22 | **0.62** | 0.37 | 0.00 |
| 23 | **0.77** | 0.15 | 0.01 |
| 24 | **0.60** | 0.37 | -0.00 |
| 25 | **0.57** | 0.25 | -0.08 |
| 26 | -0.35 | 0.39 | 0.53 |
| 27 | 0.26 | -0.10 | **0.77** |
| 28 | 0.26 | -0.08 | **0.83** |
| 29 | 0.08 | -0.07 | **0.81** |
| 30 | -0.38 | 0.22 | **0.77** |

Note. Table displays pattern coefficients using polychoric correlations from factor analysis with promax rotation. Items 26-30 were reverse-scored. Items 7, 9, 11, and 26 were initially removed. Item 10 was removed after repeating the factor analysis with 26 items, resulting in a 25-item scale.

Supplemental Table S5. Confirmatory factor analyses to test for measurement invariance across participants with one or multiple health conditions and across health conditions categories

We conducted multigroup Confirmatory Factor Analyses (MCFAs) for different numbers of health conditions and various health condition categories. Tables S5a and S5c present the tests for each step of the measurement invariance analyses of the I-HEARTS scale's three-factor model across the number of health conditions and different health condition categories. Initially, we established baseline models without equality constraints. Based on the pattern of fit indices, both sets of MCFAs demonstrated good configural model fit. Furthermore, for the MCFA across the number of health conditions, most of the standardized loadings exceeded 0.60 (all p-values < 0.001). For the MCFA across different health condition categories, most standardized loadings exceeded 0.40 (all p-values < 0.05). Tables S5b and S5d display the comparison of measurement invariance models. The results indicate strict measurement invariance for the I-HEARTS scale's three-factor model across both the number of health conditions and different health condition categories.

## Table S5a. Configural, weak, strong, and strict invariance models for I-HEARTS Scale three-factor model for participants with one, two, or more than two health conditions

| Model | Scaled  Chi-square | df | p | Robust RMSEA [90% CI] | Robust CFI | SRMR |
| --- | --- | --- | --- | --- | --- | --- |
| Configural | 1,077.260 | 816 | 0.000000001830478 | 0.062871 [0.052213, 0.072815] | 0.9818974 | 0.07706554 |
| Weak | 1,033.344 | 860 | 0.000040118546889 | 0.064259 [0.047977, 0.078438] | 0.9800698 | 0.08916893 |
| Strong | 1,081.831 | 904 | 0.000038889006496 | 0.063495 [0.047463, 0.07744] | 0.9795453 | 0.09060471 |
| Strict | 1,129.673 | 954 | 0.000068513804457 | 0.062543 [0.046169, 0.076649] | 0.9790566 | 0.09344113 |

Table S5b. Tests of measurement invariance models for I-HEARTS Scale three-factor model for participants with one, two, or more than two health conditions

| Model comparison | Delta Chi-square | Delta df | p | Delta RMSEA | Delta CFI | Delta SRMR |
| --- | --- | --- | --- | --- | --- | --- |
| Configural vs. Weak | 49.62392 | 44 | 0.259095 | 0.001388 | -0.001828 | 0.012103 |
| Weak vs. Strong | 58.86385 | 44 | 0.066294 | -0.000764 | -0.000524 | 0.001436 |
| Strong vs. Strict | 59.28243 | 50 | 0.173077 | -0.000952 | -0.000489 | 0.002836 |

Table S5c. Configural, weak, strong, and strict invariance models for I-HEARTS Scale three-factor model for participants with obesity, diabetes, skin disease, HIV, chronic pain, or cancer

| Model | Scaled  Chi-square | df | p | Robust RMSEA [90% CI] | Robust CFI | SRMR |
| --- | --- | --- | --- | --- | --- | --- |
| Configural | 2,082.333 | 1,632 | 0.0000000000001838529 | 0.060493 [0.052356, 0.068146] | 0.9850211 | 0.07678532 |
| Weak | 1,978.704 | 1,742 | 0.0000583622636960435 | 0.058508 [0.043585, 0.071181] | 0.9850438 | 0.09086687 |
| Strong | 2,118.990 | 1,852 | 0.0000131668279295116 | 0.060144 [0.046194, 0.072202] | 0.9831976 | 0.09403857 |
| Strict | 2,239.585 | 1,977 | 0.0000297674027723849 | 0.058727 [0.04444, 0.070935] | 0.9828990 | 0.09681732 |

Table S5d. Tests of measurement invariance models for I-HEARTS Scale three-factor model for participants with obesity, diabetes, skin disease, HIV, chronic pain, or cancer

| Model comparison | Delta Chi-square | Delta df | p | Delta RMSEA | Delta CFI | Delta SRMR |
| --- | --- | --- | --- | --- | --- | --- |
| Configural vs. Weak | 109.9607 | 110 | 0.483123 | -0.001986 | 0.000023 | 0.014082 |
| Weak vs. Strong | 218.6344 | 110 | 0.000000 | 0.001636 | -0.001846 | 0.003172 |
| Strong vs. Strict | 136.0032 | 125 | 0.236271 | -0.001418 | -0.000299 | 0.002779 |

Supplemental Table S6. Item-level item-total correlations for 25-item I-HEARTS Scale

| Item | Item-total correlation |
| --- | --- |
| 1 | 0.79 |
| 2 | 0.75 |
| 3 | 0.61 |
| 4 | 0.76 |
| 5 | 0.71 |
| 6 | 0.56 |
| 8 | 0.67 |
| 12 | 0.77 |
| 13 | 0.73 |
| 14 | 0.79 |
| 15 | 0.80 |
| 16 | 0.64 |
| 17 | 0.84 |
| 18 | 0.65 |
| 19 | 0.65 |
| 20 | 0.88 |
| 21 | 0.87 |
| 22 | 0.84 |
| 23 | 0.83 |
| 24 | 0.82 |
| 25 | 0.67 |
| 27 | 0.59 |
| 28 | 0.63 |
| 29 | 0.45 |
| 30 | 0.24 |

Note. Polychoric correlations are presented. Items 27-30 were reverse-scored.

Supplemental Table S7. Mean scores on psychosocial measures (*N*=300)

|  | Mean | Standard Deviation |
| --- | --- | --- |
| Internalized Shame Scale |  |  |
| Shame | 38.54 | 23.39 |
| Self-Esteem | 15.16 | 5.73 |
| UCLA Loneliness Scale | 48.36 | 13.41 |
| IPQ - Emotional | 18.19 | 6.50 |
| PHQ-9 | 8.94 | 6.77 |
| GAD-7 | 7.18 | 6.01 |
| Social Anxiety Disorder | 9.11 | 9.95 |
| CDC Healthy Days |  |  |
| Self-rated health | 3.16 | 0.96 |
| Unhealthy days, physical | 10.10 | 10.56 |
| Unhealthy days, mental | 11.44 | 10.82 |
| Total unhealthy days | 16.88 | 11.86 |
| WHODAS 2.0 | 24.54 | 9.71 |
| General Self-Efficacy | 30.37 | 5.74 |
| Perceived Stress Scale | 6.61 | 3.98 |

Note. Of the total number of participants, 41.7% (n=125) scored a 10 or above on the PHQ-9, indicating moderate to severe depression; 30.7% (n-92) scored a 10 or above on the GAD-7, indicating moderate to severe anxiety. IPQ=Revised Illness Perceptions Questionnaire (Emotional Representations subscale); PHQ-9 = Patient Health Questionnaire-9; GAD-7 = Generalized Anxiety Disorder-7; CDC=Centers for Disease Control; WHODAS=World Health Organization Disability Assessment Schedule.
